# Personal

**Published:** 1896-03

**Authors:** 


### Personal.

DR. JAMES D. SPENCER, of Watertown, was elected president of the Medical Society of the State of New York at its last annual meeting. Dr. Spencer is one of the most prominent physicians in the state. He has been a member of the society for ten years, has been a loyal supporter of all the reforms it has inaugurated, and a regular attendant at its meetings. Dr. Spencer's father was vice-president of the society in 1883, and is still an active physician. The society has done itself distinguished honor in this election.

---

DR. F. G. NOVY, of Michigan University, the distinguished bacteriologist and, with Vaughn, author of Leucomaines and Ptomaines, will read a paper before the Microscopical Society in the Buffalo Library building, Monday evening, March 9, 1896, at 8.30, on Bacterial Toxins and Antitoxins. The officers of the microscopical club extend a cordial invitation to the medical profession to hear Dr. Novy and thus signify their appreciation of his visit to Buffalo.

Dr. Daniel Lewis, of New York, president of the state board of health, has resigned the editorship of the *American Medical Review*. This must be a severe blow to this new journal, that started out so propitiously a few months ago.

---

Dr. Max Keiser, of Buffalo, recently interne at the Sisters of Charity Hospital, has gone to Europe and will devote himself to hospital work and medical study at Vienna during the next three years.

